# Comparative Small RNA Profiling and Functional Exploration on Wheat With High- and Low-Cadmium Accumulation

**DOI:** 10.3389/fgene.2021.635599

**Published:** 2021-04-15

**Authors:** Yuqing Liu, Xudong Wang, Leyi Yuan, Yuxiang Liu, Tong Shen, Yunhua Zhang

**Affiliations:** Anhui Province Key Laboratory of Farmland Ecological Conservation and Pollution Prevention, School of Resources and Environment, Anhui Agricultural University, Hefei, China

**Keywords:** wheat, cadmium accumulation, small RNA sequencing, GO and KEGG pathway analysis, microRNA profiling patterns

## Abstract

Cadmium is a toxic metal widely found in workplaces and plant soil because of extensive industrialization. Wheat is an important source of food generated from plant soil. The different responses of wheat against different omic levels of cadmium have been observed and widely studied worldwide. With the development of high-throughput sequencing, micro-level biological research has extended to the microRNA level. In this study, high-cadmium-accumulating wheat cultivars (Annong9267) and low-cadmium-accumulating wheat cultivars (Qian 102032) were used as experimental models. The two cultivars were treated by Cd for 2 h to explore the microRNA profiles in root and leaf tissues through small RNA sequencing. Important small RNAs, such as tae-miR9663-5p and tae-miR6201, and potential small RNA-mediated mechanisms associated with cadmium accumulation were identified by summarizing specific microRNA profiling patterns and their respective target genes. At the wheat roots and leaves, differentially expressed small RNAs related to cadmium accumulation in different plant tissues (roots or leaves) were identified, and functional enrichment analyses on target genes of differentially expressed miRNAs in low- and high-cadmium-accumulating wheat cultivars in different plant tissues (roots or leaves) obtained some known mature miRNAs and new miRNAs. The identified miRNA will be regarded as a potential screening biomarker for low-cadmium-accumulating wheat.

## Introduction

Heavy metal pollution caused by extensive industrialization, urbanization, and improper wastewater treatment can affect the physical processes of plants (Ashraf et al., 2019, 2020; Fischer et al., 2020). Approximately 2.35 × 10^12^ m^2^ of arable land worldwide is contaminated by heavy metals (Li et al., 2017). Among the nonessential heavy metals, cadmium (Cd) is perhaps the metal that has attracted the most attention. Cd concentrations in durum wheat grain harvested in some areas of the northern Great Plains in the United States and adjoining regions of Canada have been reported to exceed 100 μg kg^–1^ dry weight (Guo et al., 2010). In China, approximately 2.786 × 10^9^ m^2^ of agricultural soil was polluted with Cd (Li et al., 2017). Several cereals uptake cadmium from soil and water and accumulate this metal in their shoots and edible parts. In general, cadmium enters from the root and then transfers to the stem, leaves, and grains by metal transporters. Wheat, a conventional and worldwide staple food source that accounts for 30% of calorie consumption, can also uptake cadmium and often exceeds the maximum standard exposure level (Alaux et al., 2018). Cadmium concentration in durum wheat grain harvested on Canadian prairies has reached 300 μg kg^–1^, thereby exceeding the limit set by the Codex Alimentarius Commission (200 μg kg^–1^) (Chen et al., 2018). Decreasing the cadmium uptake in wheat to ensure grain quality and improve wheat growth is necessary for human health. Thus, the interaction between cadmium and wheat must be thoroughly understood.

Investigating microRNAs (miRNAs) can provide insights into plant biological processes. MiRNAs are a group of endogenous, single-stranded, short non-coding RNAs with partially self-complementary stem–loop structures that play key roles in gene expression regulation, predominantly in post-transcription via cleavage or translational repression of target mRNAs. In plants, these molecules regulate cellular differentiation, development, and metabolism, which are involved in responses to environmental changes, such as light, nutrition, temperature, and abiotic and biotic stresses (Axtell and Meyers, 2018). Several miRNAs actively play roles in wheat biological development. Debernardi et al. (2017) proved that miRNA172 plays a crucial part in wheat spike morphogenesis and grain threshold. Zhao et al. (2016) found that tae-miR408 can regulate the heading time in wheat via controlling the TaTOC1 gene. Guo et al. (2018) showed that miR9678 affects seed germination in wheat. Han et al. (2014) found that miR164 and miR169 show different expression patterns during seeding, suggesting that they perform different kinds of regulation in various developmental stages of wheat seeding. With high-throughput sequencing, different groups of miRNAs are found to participate in different biological processes, such as cell division, carbohydrate metabolism, and stress response. Meng et al. (2013) identified 104 miRNAs that are potentially involved in grain filling. Ravichandran et al. (2019) identified 104 miRNAs that regulate heat stress response, 36 of which are differentially expressed after heat stress. For metal toxicity response, Wang et al. (2018) found that miR319 can regulate cadmium, mercury, aluminum, and manganese uptake in *Medicago truncatula*. Zhou et al. (2012) reported that miR393 and miR396 regulate cadmium, mercury, and aluminum uptake in *Brassica napus*. Zhou et al. (2019) identified several miRNAs targeting heavy metal ATPases, which are responsible for Cd root-to-shoot translocation. Zhou et al. (2017) identified and characterized miRNAs in two cultivars: low (SJ19) and high-Cd-accumulating cultivars (CX4) of *Brassica parachinensis*. However, miRNAs related to wheat cadmium uptake remain to be explored.

Understanding wheat responses to cadmium will help to decrease the toxic metal uptake, thereby increasing food safety and quality. However, the yield penalty for harboring low cadmium in wheat and acreage either in China or in other countries, which grows low-cadmium accumulators, has not been reported. Based on field trials and cluster analysis in previous studies, 60 wheat cultivars were preliminary screened and divided into three categories: low-Cd accumulators, middle-Cd accumulators, and high-Cd accumulators in grain. In the following studies, the high-Cd accumulator (Qian 102032) and low-Cd accumulator (Annong9267) were verified again. The high- and low-Cd-accumulating wheat types have good reproducibility and stability in the annual accumulation, which are suitable for later cultivation and variety identification. In this work, two different cadmium-accumulating wheat (*Triticum aestivum* L.) cultivars, namely, Annong9267 and Qian 102032, were used as plant materials to identify miRNAs that respond to cadmium uptake. Then, next-generation miRNA sequencing was applied to profile the different responses of miRNA expression in low- and high-cadmium-accumulating wheat cultivars. Known and novel miRNAs associated with cadmium accumulation were identified, and their functions were analyzed to understand the genes related to cadmium uptake. The findings provided insights into the regulation of cadmium uptake and an opportunity to improve grain quality and reduce food cadmium contamination.

## Materials and Methods

### Wheat Growth Conditions and Cadmium Treatment

Two different cadmium-accumulating wheat (*T. aestivum* L.) cultivars, namely, Annong9267 (low-cadmium accumulator) and Qian 102032 (high-cadmium accumulator), were used as plant materials. Uniform seeds of wheat were surface sterilized in 10% NaClO and germinated at 25°C for 3 days in the dark. The germinated seeds were grown in Petri dishes (diameter 18 cm, each containing 50 seeds) floating on half-strength 800 mmol m^–2^ s^–1^ Hoagland’s solution in a growth chamber under a 12 h photoperiod, 70% relative humidity, and 25°C/18°C (day/night). The plants were grown hydroponically for 10 days and then transferred to the same nutrient solution containing 200 mmol L^–1^ CdCl_2_.

Based on previous studies, after 2 h of cadmium treatment, the content of cadmium accumulated in the two cultivars reached the peak. The leaves and roots of the seedlings treated with 200 mmol L^–1^ of CdCl_2_ hydroponic culture for 2 h on the 10th day of growth were separately harvested, immediately frozen in liquid nitrogen, and stored at 80°C. Six root samples and six leaf samples were collected and classified into four groups for future small RNA sequencing: Group 1 (samples 1–3), Annong9267 leaf samples; Group 2 (samples 4–6), Annong9267 root samples; Group 3 (samples 7–9), Qian 102032 leaf samples; and Group 4 (samples 10–12), Qian 102032 root samples. Each group contained three replications.

### RNA Extraction

The frozen root and leaf samples were subjected to RNA extraction. Total RNA samples were extracted using the mirVana miRNA Isolation Kit (Cat#. AM1561, Austin, TX, United States) following the manufacturer’s protocol. RNA integrities were evaluated using the Agilent 2100 Bioanalyzer (Agilent Technologies, Santa Clara, CA, United States), and all qualified RNA samples should meet the criteria of RNA integrity number (RIN) ≥ 7.0.

### Small RNA Library Construction

Ligate 3′ was added following the general workflow for small RNA library construction. In brief, 1 μg of total RNA was subjected to connect 3′ adapter, heated using a polymerase chain reaction (PCR; Applied biosystems, 9700) instrument at 70°C for 2 min, added to a ligation buffer, RNase inhibitor, and T4 RNA Ligation2, mixed, and reacted at 25°C for 1 h. Stop solution was added and reacted at 28°C for 15 min. The solution was immediately placed on ice, and 5′ adapters were added. The 5′ adapter mix system was placed in a PCR instrument at 70°C for 2 min and then added into the solution to react at 25°C for 1 h. After the reaction, the solution was immediately placed on ice.

For reverse transcription-polymerase chain reaction (RT-PCR), First-strand Master Mix, Super Script II (Invitrogen), dNTP mix, and 5′ and 3′ adapter RNA and RNase inhibitors were mixed and heated at 50°C for 1 h. PCR Primer Cocktail and Master Mix were then added, and the solution was subjected to 98°C for 30 s, followed by 11 cycles of 98°C for 10 s, 60°C for 30 s, and 72°C for 15 s, maintained at 72°C for 10 min, and held at 4°C. The PCR products were purified by electrophoresis, and the gel stripe between 147 and 157 nt was cut off and recollected. The DNA was precipitated and dissolved in 10 μL of 10 mM Tris-HCl (pH 8.5).

The library was quantified by a Qubit 2.0 Fluorometer, and its quality was validated using an Agilent Technologies 2100 Bioanalyzer (Agilent Technologies). Cluster generation was then conducted, and 10 μL of 2 nM template DNA was subjected to cluster generation, hybridized to a paired end flow cell, and amplified by bridge amplification on the Illumina cBot. The flow cells were then loaded onto the HiSeqTM 2500 platform and sequenced.

### Small RNA Sequencing and Analysis

Base calling was conducted to convert raw reads into sequence data. Impurities in raw data were then filtered. Reads with low quality and reads shorter than 10 nt, 5′ primer contaminants and ploy (A), and reads without 3′ adapter and insert tag were eliminated. After filtering, the remaining clean reads were stored in FASTQ format.

Clean reads with lengths between 18 and 40 nt were then mapped with wheat genome [international wheat genome sequencing consortium (IWGSC), version number miRBaseV22] (Wang et al., 2014) and annotated with miRBase^1^ to identify known miRNAs using Bowtie software (version: v0.12.7) (Griffiths-Jones et al., 2007; Langmead and Salzberg, 2012). For novel miRNAs, the unannotated reads that could be aligned to the genome were analyzed using miRCat (version: V4.4.Alpha.D) (Moxon et al., 2008). When analyzing the folding model, if the sequence is located in the stem loop structure, then it is preliminarily determined as a new miRNA candidate.

The expression level of known annotated miRNAs and novel miRNAs was analyzed. The number of reads for each miRNA was normalized by the trimmed mean of M values (TMM) and then converted into transcripts per million (TPM, the number of reads on a miRNA × 10^6^/total read number) to standardize the expression of miRNA and allow the comparison for miRNA expression levels among different miRNAs and samples.

Differentially expressed miRNAs (DEMs) were identified by edgeR (version: v3.2.5) analysis and calculated *p*-values. After *p*-value calculation, multiple-hypothesis testing, and correction, the *p*-value threshold was determined by controlling the false discovery rate (FDR) to obtain the corrected *p*-value, namely, *q*-value. Multiples of differential expression were also calculated on the basis of the TPM value to obtain fold change. In the analysis, the *p*-value threshold was set to ≤0.05, and the fold change was set to ≥2. MiRanda (version: v3.3a) was used to predict whether the miRNA could bind to the 3′ UTR of mRNAs to identify the targets of these DEMs (Turner, 1985).

### Gene Ontology and KEGG Pathway Analysis

The differentially expressed miRNA target genes were analyzed by Gene Ontology (GO) enrichment and Kyoto Encyclopedia of Genes and Genomes(KEGG) pathway enrichment using R studio based on the hypergeometric distribution (Kanehisa and Goto, 2000; Consortium, 2004). Target genes corresponding to the three levels of the biological process, namely, cellular component and molecular function, were first counted for GO enrichment analysis. The specific principle is to map the target genes of different miRNAs to the terms of the GO database, calculate the number of genes in each entry, and apply the hypergeometric test to screen for significantly enriched target genes compared with the entire genome background. For GO entries, the calculation formula is presented as follows:

$$P=1-\sum_{\text{i}=0}^{m-1}\frac{\left(\begin{array}{c} M\\ i \end{array}\right)\,\left(\begin{array}{c} N-M\\ n-i \end{array}\right)}{\left(\begin{array}{c} N\\ n \end{array}\right)},$$

where *N* is the number of genes annotated with GO in all genes, *n* is the number of target genes in *N*, *M* is the number of genes annotated as a specific GO term in all genes, and *m* is the number of target genes annotated as a specific GO term. After the calculated *p*-value was corrected by multiple hypothesis testing, *q* ≤ 0.05 was used as the threshold. GO terms that met this condition were defined as significantly enriched in the differentially expressed genes.

For GO enrichment analysis, emphasis was given for the rich factor, which was calculated as follows: (the number of target genes in a GO term/the number of all target genes correspond to the GO database)/(the number of genes contained in a GO term/the total number of genes correspond to the GO database). A high rich factor indicated a high degree of enrichment. *Q*-value was the corrected *p*-value after multiple hypothesis testing correction: when the *Q*-value is small, the enrichment is significant.

KEGG was used to identify significantly enriched metabolic or signal transduction pathways in DES target genes. The KEGG enrichment formula was similar to that of GO analysis. *p*-value was corrected by the Bonferroni method, and the threshold was set to *q*-value ≤ 0.05. KEGG terms that met this condition were defined as significantly enriched.

## Results

### Deep Sequencing of Small RNA in Different Cadmium-Accumulating Wheat Cultivars

The roots and leaves from two different cadmium-accumulating wheat cultivars, Annong9267 (low-cadmium accumulator) and Qian 102032 (high-cadmium accumulator), were collected, and 12 sRNA libraries were created and sequenced to investigate different sRNA profiles in different cadmium-accumulating wheat cultivars.

The sequencing results showed that 19,755,465 to 48,544,084 raw reads were generated per library (Table 1). After pre-processing, low-quality reads were removed. A total of 18,630,273 to 48,056,605 clean reads were filtered out from each library. The clean ratio ranged from 94.3 to 99.6%, thereby showing excellent sequencing quality.

**TABLE 1:** Statistics of sequencing reads.

Sample ID*	**Raw reads**	**Clean reads**	**Clean ratio (%)**
1	26,325,961	26,131,399	99.26
2	29,898,759	29,648,807	99.16
3	48,544,084	48,056,605	99.00
4	19,755,465	18,630,273	94.30
5	35,690,273	34,563,303	96.84
6	33,262,949	32,494,875	97.69
7	28,735,748	28,621,389	99.60
8	29,612,598	29,107,455	98.29
9	39,719,147	38,995,735	98.18
10	27,328,289	26,527,183	97.07
11	39,844,116	39,272,009	98.56
12	22,711,156	21,861,060	96.26

*samples 1–3 Annong9267 leaf, samples 4–6 Annong9267 root, samples 7–9 Qian 102032 leaf, samples 10–12 Qian 102032 root.

### Annotation of Small RNAs

**FIGURE 1:**
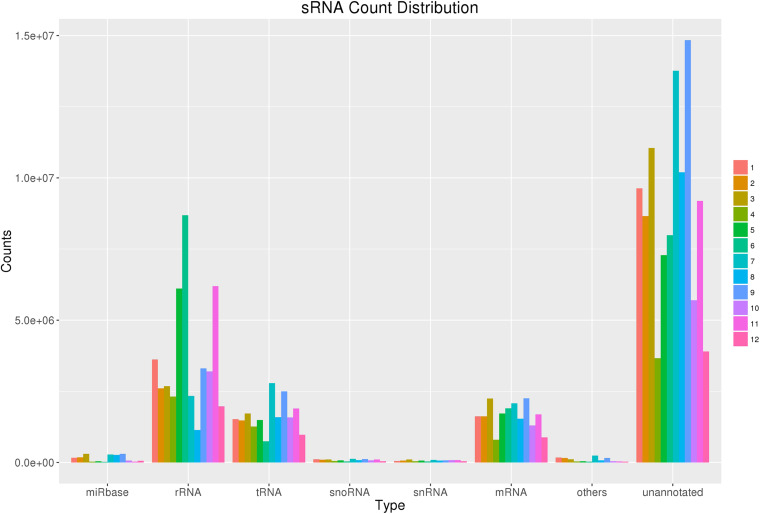
sRNA count distribution. Count distribution of different RNA categories in all 12 samples from four groups. sRNAs were categorized into multiple groups according to their biological classification. The first category “miRbase” indicates annotated some RNAs using the miRbase database.

After filtering, the clean reads were mapped to IWGSC and annotated by miRBase. Figure 1 and Table 2 show the distribution of catalogs of small RNA of mapped reads. Several known mature miRNAs and a number of unannotated reads were successfully obtained. This result would lead to the further identification of novel miRNAs. A total of 126 known mature miRNAs (Supplementary Table 1) and 63 pri-miRNAs (Supplementary Table 2) were identified.

**TABLE 2:** Statistics of mapped reads.

Sample ID*	**All reads**	**miRbase**	**rRNA**	**tRNA**	**snoRNA**	**snRNA**	**mRNA**	**Others**	**Un-annotated**
1	16,905,829	166,095	3,620,978	1,524,571	115,919	52,800	1,620,167	171,246	9,634,053
2	14,867,094	17,9522	2,604,021	1,474,035	100,342	66,736	1,623,296	161,186	8,657,956
3	18,338,608	303,373	2,680,893	1,724,228	105,320	108,288	2,245,241	115,840	11,055,425
4	8,210,721	28,491	2,316,637	1,265,750	54,635	44,479	797,151	36,479	3,667,099
5	16,842,322	47,669	6,108,621	1,490,545	72,704	66,647	1,721,547	47,014	7,287,575
6	19,457,739	22,099	8,688,528	749,209	39,966	38,849	1,900,782	27,979	7,990,327
7	21,717,532	280,580	2,337,620	2,789,033	128,234	87,193	2,083,463	246,605	13,764,804
8	14,954,364	265,876	1,142,975	1,592,862	80,608	65,971	1,537,065	71,660	10,197,347
9	23,564,808	302,536	3,308,267	2,498,423	124,016	78,042	2,252,947	157,209	14,843,368
10	12,054,573	71,056	3,199,430	1,583,579	73,587	82,284	1,300,087	41,658	5,702,892
11	19,231,928	31,250	6,196,039	1,897,626	102,386	83,912	1,693,828	37,162	9,189,725
12	7,913,054	57,466	1,972,023	976,299	44,726	46,225	886,495	26,292	3,903,528

*samples 1–3 Annong9267 leaf, samples 4–6 Annong9267 root, samples 7–9 Qian 102032 leaf, samples 10–12 Qian 102032 root.

### Prediction of Novel Small RNAs

After annotation, the unannotated tags and predicted novel miRNAs were further analyzed by miRCat. A total of 751 novel miRNAs were predicted (Supplementary Table 5). Most of the novel miRNAs had a relatively low expression, but some had great abundance. In comparison with root small RNAs (Group 2 vs. Group 4), miRNAs such as novel.504 (only detected in group 2, *p*-value of 1.87E-19 and FDR of 3.30E-17) and novel.411 (log2 transformed fold change of 2.077441, *p*-value of 9.97E-06, and FDR of 0.00706) exhibited differential expression levels in the two aforementioned groups. In comparison with the leaves (Group 1 vs. Group 3), novel.526 was differentially expressed with log2 transformed fold change of 3.137454561, *p*-value of 5.30E-09, and FDR of 2.19E-07. These results indicated that these novel miRNAs were conservatively expressed in certain wheat cultivars, which might play important roles in cadmium uptake regulation.

### DEMs in Different Cadmium-Accumulating Wheat Cultivars

Transcripts per million was calculated to indicate relative abundance and make the expression levels comparable for the identification of DEMs in different cadmium-accumulating wheat cultivars. The following two comparisons were performed: Group 1 with group 3 (group 1/3) and group 2 with group 4 (group 2/4). The threshold and fold change were set to *p*-value ≤0.05 and ≥2, respectively, to determine the significant changes in miRNA expression in different cultivars.

**FIGURE 2:**
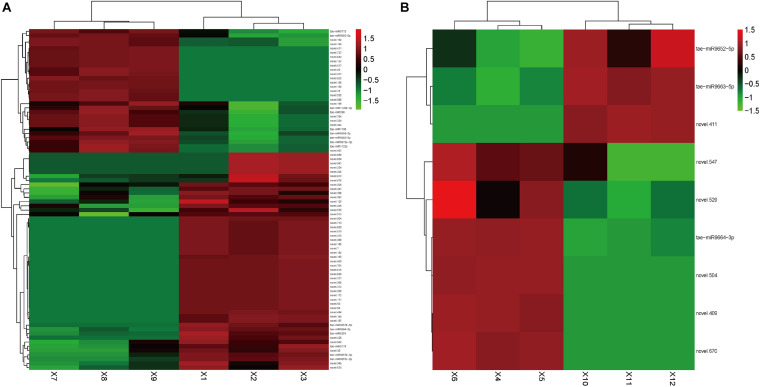
Heatmap of differentially expressed small RNAs. Heatmap presenting the differentially expressed small RNAs in two tissues of the wheat, **(A)** roots and **(B)** leaves, in which green indicates down-regulation, and red indicates up-regulation.

In Group 1/3, 80 DEMs were identified (Supplementary Table 3). Among them, 51 miRNAs were upregulated, and tae-miR9664-3p, tae-miR6201, and Novel.504 were the most abundant. Tae-miR9657c-3p (*p*-value: 5.34E-06, FDR: 0.000140097), tae-miR9657b-5p (*p*-value: 1.43E-05, FDR: 0.000304258), and tae-miR9657b-3p (*p*-value: 3.51E-05, FDR: 0.00064212) also showed large fold changes, indicating that this miRNA family might play a crucial regulation role in cadmium accumulation. Among the 29 downregulated miRNAs, tae-miR9773 and tae-miR398 were the most abundant. Tae-miR9663-5p and tae-miR9655-3p showed large fold changes of -3.86 and -2.70, respectively. Figure 2A shows the heatmap of the expression level of DEMs in Group1/3.

In Group 2/4, nine DEMs were identified (Supplementary Table 4). Among them, six miRNAs were upregulated, of which tae-miR9664-3p was the most abundant. Novel.574 showed the largest fold change of 4.79. Three miRNAs were downregulated, of which tae-miR9663-5p was the most abundant, which showed the largest fold change of -3.58. Tae-miR9663-5p was expressed in the roots (with log2 transformed fold change of -3.58114 in the leaves, *p*-value of 2.41E-15, and FDR of 2.85E-13) and leaves (with log2 transformed fold change of -3.85717031, *p*-value of 8.84E-25, and FDR of 1.74E-22), and it showed similar expression change pattern in the two comparison groups. This finding indicated the potential downregulation role in cadmium uptake regulation. Figure 2B shows the heatmap of the expression level of DEMs in Group2/4. The two top candidates (novel.504 and novel.409) were expressed in both the leaves and roots of group 1.

### Target Gene Functions of DEMs in Different Cadmium-Accumulating Wheat Cultivars

Some target genes of DEMs were also identified and subjected to gene function analysis. Thousands of genes targeted by differentially expressed miRNAs were previously determined; however, analyzing their individual target gene function and biological effects would be impossible and laborious. Here, GO and KEGG terms were applied to describe the functional distribution of these targeted genes and understand the detailed correlations between the targeted genes of DEMs and their responsibility in cadmium uptake regulation.

Gene Ontology assignments identified 3318 possible target genes for 89 DEMs. The functions were analyzed according to three categories: “biological process,” “cellular component,” and “molecular function.” GO functional classifications are listed to illustrate the distribution of gene functions from the macro and biological effect levels.

**FIGURE 3:**
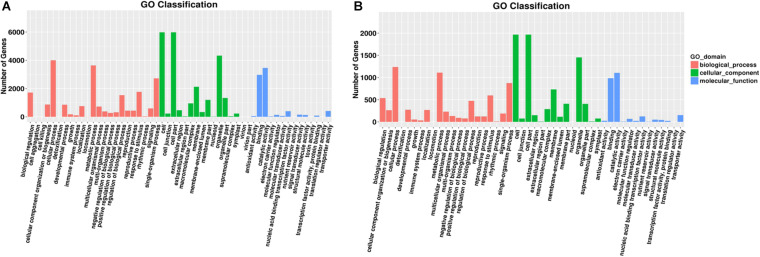
Gene number distribution in different GO classification terms. A group of significant GO terms were identified through GO enrichment analyses. Distribution of gene numbers in different GO classification terms in different tissues: **(A)** roots and **(B)** leaves. Different colors indicate different GO domains.

In group 1/3, all target genes were largely distributed in nine GO terms (Figure 3A), of which the most frequent were “cell,” “cell part,” and “organelle” all clustered in the “cellular component” category. In “biological process,” the most frequent terms were “cellular process” and “metabolic process.” In “molecular functions,” the most frequent terms were “binding” and “catalytic activity.”

In group 2/4, target genes were largely distributed in nine GO terms (Figure 3B). Similar to root samples, the most frequent terms were all clustered in the “cellular component” category. In “biological process,” “single–organism process” also showed high frequency. In “molecular functions,” the most frequent terms were “binding” and “catalytic activity,” similar to that in group 1/3 results.

**FIGURE 4:**
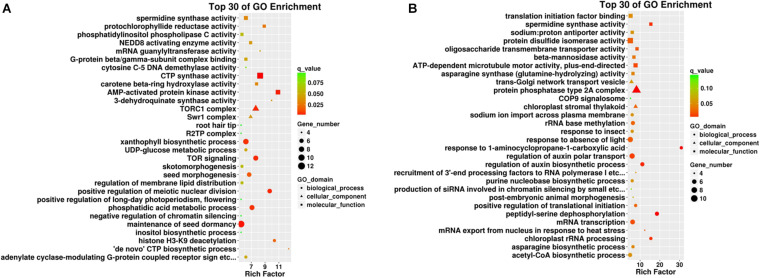
Top 30 GO enrichment terms in different plant tissues such as **(A)** roots and **(B)** leaves. The x-axis indicates the rich factor of such GO terms in each tissue. The color of the point indicates the q-value of the GO terms, the size of the point indicates the number of genes enrich in such GO term, and the shape of the point indicates the domains of the GO terms.

Gene Ontology enrichment analysis was conducted to understand the details of target gene functions. The top 30 enrichment terms of each comparison are shown in Figure 4. The rich factors were “AMP-activated protein kinase activity” and “histone H3–K9 deacetylation” in the root samples and “response to 1-aminocyclopropane-1-carboxylic acid” and “peptidyl-serine dephosphorylation” in the leaf samples. The term “spermidine synthase activity” was shared in both tissues, indicating its possible role in Cd uptake. Two major GO terms were differently enriched as “TORC1 complex” (significant in root samples) and “response to 1-aminocyclopropane-1 carboxylic acid” (significant in leaf samples), and one GO term was significantly shared and enriched as “spermidine synthase activity” in both tissues.

**FIGURE 5:**
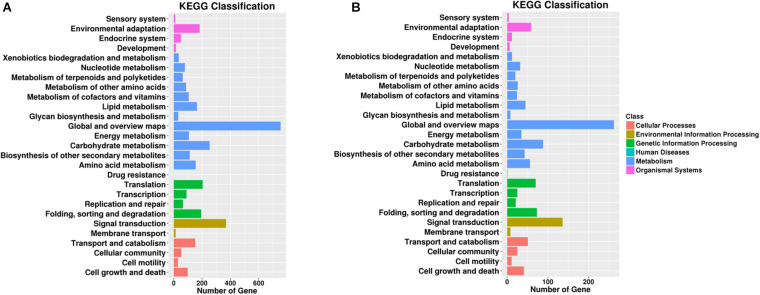
Gene number distribution in different KEGG classification terms. Top KEGG terms in **(A)** roots and **(B)** leaves. The x-axis indicates the number of genes enriched in such KEGG term, and the colors of the bar indicate different KEGG classes including cellular processes and environmental information processing.

**FIGURE 6:**
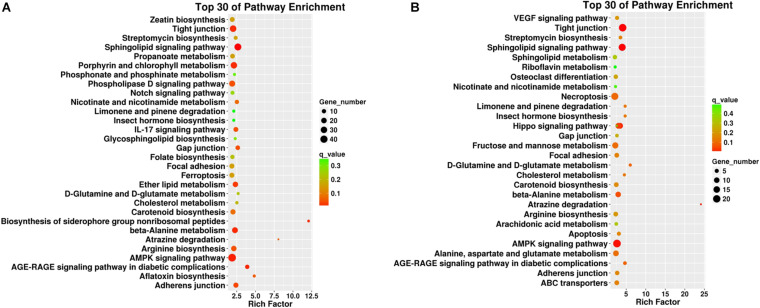
Top 30 pathway enrichment results from the KEGG analyses in different plant tissues such as **(A)** roots and **(B)** leaves. The x-axis indicates the rich factor of such KEGG terms in each tissue. The color of the point indicates the q-value of the KEGG terms, and the size of the point indicates the number of genes enrich in such KEGG term.

Pathway enrichment analysis based on KEGG database and generated reports for target genes in each pairwise was conducted to understand the interactions among target genes. The functions were analyzed according to six categories: “cellular processes,” “environmental information processing,” “genetic information processing,” “human diseases,” “metabolism,” and “organismal systems.” KEGG classifications are listed to illustrate the distribution of pathway functions at the biological effect level. A bar plot for the statistics of KEGG term classification is shown in Figure 5, and a scatter plot to display the top 20 KEGG enrichment results is illustrated in Figure 6.

For KEGG term classification, the two comparisons showed similar results. The terms with most abundant gene numbers were “environmental adaptation,” “global and overview maps,” “carbohydrate metabolism,” “translation,” “folding, sorting, and degradation,” and “signal transduction.”

For KEGG enrichment analysis, the richest factors were “biosynthesis of the siderophore group non-ribosomal peptides,” “atrazine degradation” in root samples, and “atrazine degradation” in leaf samples. “Atrazine degradation” was shared in both tissues, indicating that the regulation of Cd uptake and atrazine degradation might share some common steps in the roots and leaves. The “AMPK signaling pathway” is a top shared pathway in both tissues. In particular, it was found that “porphyrin and chlorophyll II metabolism” were enriched in the roots, and “necroptosis” was enriched in the leaves, indicating potential tissue-specific cadmium intake and accumulation-associated pathways.

## Discussion

A comprehensive analysis was conducted on the miRNA expression patterns in low- and high-cadmium-accumulating wheat cultivars with tissue-specific exploration on the roots and leaves by using small RNA sequencing techniques. New small RNA properties associated with cadmium accumulation and tissue specificity in wheat were identified at two major levels: (1) differentially expressed small RNAs associated with cadmium accumulation in different plant tissues (roots or leaves) and (2) functional enrichment analyses on target genes of DEMs in low- and high-cadmium-accumulating wheat cultivars in different plant tissues (roots or leaves). These results were validated on the basis of previously reported publications in wheat.

### Differentially Expressed Small RNAs in Different Plant Tissues

A group of functional small RNAs were differentially expressed in low- and high-cadmium-accumulating wheat cultivars either in the roots or in the leaves. In the roots, emphasis was given on the two levels of functional small RNAs with either high abundance or differentially expressed patterns comparing low- and high-cadmium-accumulating plants. Three typical small RNAs in the roots with relatively high expression level in the high-cadmium accumulation group and significant fold change comparing low- and high-cadmium-accumulating wheat cultivars were selected to filter and identify the significant small RNAs that contributed to cadmium accumulation.

In the roots, tae-miR9663-5p, a typical miRNA identified in wheat in 2014, was found to be a significant miRNA with TPM of 4158.93719 in high-cadmium-accumulating roots and 286.9844726 in low-cadmium-accumulating roots (log transformed fold change of -3.86, Supplementary Table 3). According to recent publications in 2014, this miRNA was upregulated during the initiation of wheat seeds, particularly during the pre-seedling stage, thereby confirming its functional role in wheat (Han et al., 2014). Another independent research identified this miRNA as a typical miRNA biomarker for the identification of high-cadmium-accumulating genotypes, thereby validating the present result (Zhou et al., 2020). The abovementioned research selected the root as the target plant organ for studying cadmium accumulation (Zhou et al., 2020). Therefore, the first identified biomarker, tae-miR9663-5p, is a root-specific cadmium accumulation indicator in wheat. Another upregulated miRNA in high-cadmium accumulation roots is tae-miR6201 with TPM of 661.3927583 in low-cadmium accumulation roots and 107.1818898 in high-cadmium accumulation roots (log transformed fold change of 2.625446091). According to recent publications, tae-miR6201 participates in the initiation of roots in wheat (Zhang et al., 2018; Li et al., 2019), thereby confirming its tissue specificity. For its correlations with cadmium accumulation, only a few publications have discussed the detailed functional correlations between tae-miR6201 and cadmium accumulation. Early in 2014, a systematic analysis on new wheat miRNAs with high-throughput sequencing techniques confirmed that such a miRNA was associated with the intake and metabolism of cadmium (Kurtoglu et al., 2014), thereby corresponding to our prediction. Therefore, the top miRNA, namely, tae-miR6201, may also be a cadmium accumulation-associated miRNA with root specificity.

In addition to root-specific miRNAs, a group of effective miRNAs were identified in the leaves of high- and low-cadmium accumulation groups. The number of typical miRNAs with differentially expressed levels in the leaves of high- and low-cadmium accumulation groups was lower than that in the roots. One of the significant miRNAs in the leaves had a similar fold change of -3.581, *p*-value of 2.41E-15, and FDR of 2.85E-13. Such a miRNA is functional in the roots for cadmium accumulation. However, the same publication confirming the presence of tae-miR9663-5p in wheat roots also validated a relatively high expression level of this miRNA in the flag leaves of wheat (Han et al., 2014). In 2019, an independent study (Zhou et al., 2019) on the effects of cadmium on the miRNA of the entire plant confirmed that plants with different Cd metabolism capacities might have different expression levels of typical miRNAs, including our target miRNA, namely, tae-miR9663. Therefore, as a shared typical miRNA biomarker for distinguishing high- and low-cadmium-accumulating plants in the roots and leaves, tae-miR9663-5p is an important potential biomarker for monitoring the cadmium accumulation capacity of plants in either the leaves or roots. Another top upregulated miRNA comparing low- and high-cadmium accumulating leaves is tae-miR9664-3p, which is also highly expressed in the leaves (Han et al., 2014). For its detailed correlations with cadmium intake and accumulation, tae-miR9664 is upregulated in the flag leaves of wheat (Ramachandran et al., 2020), thereby corresponding to the tissue specificity of our findings. For its correlations with cadmium accumulation, an independent study (Zhou et al., 2019) on the effects of cadmium on miRNA also identified this miRNA as a candidate cadmium intake and metabolism-associated miRNA, thereby validating our results.

A group of shared or unshared small RNAs that contributed to the regulation of cadmium metabolism and accumulation were identified. These molecules might be potential biomarkers for detecting and monitoring the cadmium accumulation status of wheat to promote the development of relevant agricultural technologies against cadmium pollution.

### Functional Enrichment Analyses on Target Genes of DEMs

A group of miRNAs that can distinguish low- and high-cadmium-accumulating plants in either the roots or leaves were identified. Given the complex role of miRNAs in gene expression regulation, a single or several DEMs cannot describe the comprehensive functional differentiation. Here, GO and KEGG pathway enrichment analyses were conducted to present the functional distribution profiling of the target genes for our candidate miRNAs. The top GO and KEGG terms in either the roots or leaves were selected for detailed analyses.

In the roots, two effective GO terms, namely, AMP-activated protein kinase activity and histone H3-K9 deacetylation, were identified as the top GO terms derived from the target genes of differentially expressed miRNAs, indicating their potential relationships with cadmium accumulation. According to recent publications, early in 2012, researchers from Poland confirmed that a specific protein kinase SNF1-related protein kinase type 2 contributed to the accumulation of cadmium in the roots via the activation of AMP-activated protein kinase activity (Kulik et al., 2012). Therefore, the target genes of significant small RNAs could be enriched in such GO terms. Furthermore, histone H3-K9 deacetylation has been widely reported to be correlated with the pathological effects of cadmium in different kinds of plants, including wheat (Zheng et al., 2016). As for KEGG enrichment results, a specific term, namely, “Atrazine degradation,” has shown to be the richest factor. Few publications discussed the relationships between Atrazine and cadmium. Only one publication in 2005 confirmed that the uptake of Atrazine might affect the accumulation rate of cadmium (Su et al., 2005). Therefore, this KEGG pathway might be one of the top enriched functional pathways, although further studies in this field were needed to reveal the detailed relationships between the two important metabolites in wheat.

Similar to the functional enrichment results in target genes of DEMs in the roots, atrazine degradation as a KEGG pathway term has also been identified in the leaves, indicating the specific relationship between atrazine and cadmium (Su et al., 2005). For GO terms, response to 1-aminocyclopropane-1-carboxylic acid and peptidyl-serine de-phosphorylation are two top terms, in which the target gene of DEMs enrich. According to early research on tomatoes (Liu et al., 2008), 1-aminocyclopropane-1-carboxylic is correlated with the improvement of cadmium tolerance, presenting a correlation between such a GO term and cadmium metabolism. Similar correlations have also been further validated in different plants, including carrot (Di Toppi et al., 1998), pea (Safronova et al., 2006), soybean (Chmielowska-Ba̧k et al., 2013), and wheat (Milone et al., 2003). Therefore, in wheat, the target genes of our candidate small RNAs are correlated with the metabolism of cadmium, thereby validating our prediction. As for peptidyl-serine de-phosphorylation, although few publications report the functional role of such biological processes in plants, in 2019, a specific study on *Caenorhabditis elegans* confirmed that such biological processes are correlated with weak cadmium stress via complex microbiological regulation, thereby validating our prediction (Dölling et al., 2019).

The top GO and KEGG enrichment terms are correlated with high- and low-cadmium accumulation in their respective plant tissues (roots or leaves), as supported by recent literature. This study identified a group of candidate miRNAs that might play a regulatory role for cadmium accumulation in different plant tissues and predicted various candidate regulatory pathways as the potential candidate biological mechanisms of cadmium accumulation for further validation.

## Conclusion

High-throughput sequencing was applied to compare the small RNA profiling of high- and low-cadmium-accumulating wheat types in two independent tissues of roots and leaves. The differentially expressed small RNAs were identified as candidate biomarkers to evaluate cadmium metabolism in roots and leaves. The target genes of differentially expressed small RNAs were identified, and such genes underwent GO and KEGG pathway enrichment to reveal the biological mechanisms for different cadmium accumulation capacities in wheat. This study selected a group of cadmium accumulation-associated small RNAs and took the initial step on revealing the biological mechanisms of cadmium accumulation in wheat. Further experimental validation and plant model-based experiments were necessary to verify the current results. In our further plans, the SNP variations of the precursor and mature miRNA sequences between high- and low-cadmium-accumulating wheat types with significant differences in cadmium accumulation will be identified to develop a biomarker for cadmium uptake.

## Data Availability Statement

The datasets presented in this study can be found in online repositories. The names of the repository/repositories and accession number(s) can be found below: NCBI (accession: PRJNA698286).

## Author Contributions

YL and YZ designed the study. YL and XW performed the experiments. LY, YL, and TS analyzed the results. YZ wrote the manuscript. All authors contributed to the research and reviewed the manuscript.

## Conflict of Interest

The authors declare that the research was conducted in the absence of any commercial or financial relationships that could be construed as a potential conflict of interest.

## Footnotes

1 http://www.mirbase.org/

## Supplementary Material

Supplementary Table 1 Counts of known mature miRNA related to wheat cadmium accumulation.

Supplementary Table 2 Counts of pri-miRNA related to wheat cadmium accumulation.

Supplementary Table 3 Differentially expressed miRNA in contrasting cadmium accumulating wheat cultivars (Group 1 vs 3).

Supplementary Table 4 differentially expressed miRNA in contrasting cadmium accumulating wheat cultivars (Group 2 vs 4).

Supplementary Table 5 Novel miRNA related to wheat cadmium accumulation.

## References

[B1] Alaux M. Rogers J. Letellier T. Flores R. Alfama F. Pommier C. (2018). Linking the international wheat genome sequencing consortium bread wheat reference genome sequence to wheat genetic and phenomic data. *Genome Biol*. 19:111. 10.1186/s13059-018-1491-4 30115101 PMC6097284

[B2] Ashraf M. A. Kumagai S. Ito K. Sugita R. Tanoi K. Rahman A. (2019). ATP binding cassette proteins ABCG37 and ABCG33 are required for potassium-independent cesium uptake in *Arabidopsis* roots. *BioRxiv* [Preprint]. 10.1101/823815 33588076

[B3] Ashraf M. A. Umetsu K. Ponomarenko O. Saito M. Aslam M. Antipova O. (2020). PIN FORMED 2 modulates the transport of Arsenite in *Arabidopsis thaliana*. *Plant Commun.* 1:100009. 10.1016/j.xplc.2019.100009 33404549 PMC7747963

[B4] Axtell M. J. Meyers B. C. (2018). Revisiting criteria for plant microRNA annotation in the era of big data. *Plant Cell* 30 272–284. 10.1105/tpc.17.00851 29343505 PMC5868703

[B5] Chen H. Zhang W. Yang X. Wang P. McGrath S. P. Zhao F. (2018). Effective methods to reduce cadmium accumulation in rice grain. *Chemosphere* 207 699–707. 10.1016/j.chemosphere.2018.05.143 29857202

[B6] Chmielowska-Ba̧k J. Lefèvre I. Lutts S. Deckert J. (2013). Short term signaling responses in roots of young soybean seedlings exposed to cadmium stress. *J. Plant Physiol.* 170 1585–1594. 10.1016/j.jplph.2013.06.019 23942356

[B7] Consortium G. O. (2004). The Gene Ontology (GO) database and informatics resource. *Nucleic Acids Res.* 32 D258–D261. 10.1093/nar/gkh036 14681407 PMC308770

[B8] Debernardi J. M. Lin H. Chuck G. Faris J. D. Dubcovsky J. (2017). microRNA172 plays a crucial role in wheat spike morphogenesis and grain threshability. *Development* 144 1966–1975. 10.1242/dev.146399 28455375 PMC5482987

[B9] Dölling R. Mendelski M. N. Paul R. J. (2019). Bacterial diet and weak cadmium stress affect the survivability of *Caenorhabditis elegans* and its resistance to severe stress. *Heliyon* 5:e01126. 10.1016/j.heliyon.2019.e01126 30705981 PMC6348244

[B10] Fischer S. Sánchez-Bermejo E. Xu X. Flis P. Ramakrishna P. Guerinot M. L. (2020). Targeted expression of the arsenate reductase HAC1 identifies cell type specificity of arsenic metabolism and transport in plant roots. *J. Exp. Bot*. 72 415–425. 10.1093/jxb/eraa465 33038235 PMC7853597

[B11] Griffiths-Jones S. Saini H. K. van Dongen S. Enright A. J. (2007). miRBase: tools for microRNA genomics. *Nucleic Acids Res.* 36 D154–D158. 10.1093/nar/gkm952 17991681 PMC2238936

[B12] Guo G. Liu X. Sun F. Cao J. Huo N. Wuda B. (2018). Wheat miR9678 affects seed germination by generating phased siRNAs and modulating abscisic acid/gibberellin signaling. *Plant Cell* 30 796–814. 10.1105/tpc.17.00842 29567662 PMC5969276

[B13] Guo X. Akhter F. Tenuta M. Flaten D. N. Gawalko E. J. Grant C. A. (2010). Mycorrhizal colonization and grain Cd concentration of field-grown durum wheat in response to tillage, preceding crop and phosphorus fertilization. *J. Sci. Food Agric.* 90 750–758. 10.1002/jsfa.3878 20355108

[B14] Han R. Jian C. Lv J. Yan Y. Chi Q. Li Z. (2014). Identification and characterization of microRNAs in the flag leaf and developing seed of wheat (*Triticum aestivum* L.). *BMC Genomics* 15:289. 10.1186/1471-2164-15-289 24734873 PMC4029127

[B15] Kanehisa M. Goto S. (2000). KEGG: kyoto encyclopedia of genes and genomes. *Nucleic Acids Res.* 28 27–30. 10.1093/nar/28.1.27 10592173 PMC102409

[B16] Kulik A. Anielska-Mazur A. Bucholc M. Koen E. Szymańska K. Żmieńko A. (2012). SNF1-related protein kinases Type 2 are involved in plant responses to cadmium stress. *Plant Physiol.* 160 868–883. 10.1104/pp.112.194472 22885934 PMC3461561

[B17] Kurtoglu K. Y. Kantar M. Budak H. (2014). New wheat microRNA using whole-genome sequence. *Funct. Integr. Genomics* 14 363–379. 10.1007/s10142-013-0357-9 24395439

[B18] Langmead B. Salzberg S. L. (2012). Fast gapped-read alignment with Bowtie 2. *Nat. Methods* 9 357–U54. 10.1038/NMETH.1923 22388286 PMC3322381

[B19] Li H. Luo N. Li Y. Cai Q. Li H. Mo C. (2017). Cadmium in rice: Transport mechanisms, influencing factors, and minimizing measures. *Environ. Pollut.* 224 622–630. 10.1016/j.envpol.2017.01.087 28242254

[B20] Li J. Jiao Z. He R. Sun Y. Xu Q. Zhang J. (2019). Gene expression profiles and microRNA regulation networks in tiller primordia, stem tips, and young spikes of wheat guomai 301. *Genes* 10:686. 10.3390/genes10090686 31500166 PMC6770858

[B21] Liu K. Shen L. Sheng J. (2008). Improvement in cadmium tolerance of tomato seedlings with an antisense DNA for 1-aminocyclopropane-1-carboxylate synthase. *J. Plant Nutr.* 31 809–827. 10.1080/01904160802043080

[B22] Meng F. Liu H. Wang K. Liu L. Wang S. Zhao Y. (2013). Development-associated microRNAs in grains of wheat (*Triticum aestivum* L.). *BMC Plant Biol.* 13:140. 10.1186/1471-2229-13-140 24060047 PMC4015866

[B23] Milone M. T. Sgherri C. Clijsters H. Navari-Izzo F. (2003). Antioxidative responses of wheat treated with realistic concentration of cadmium. *Environ. Exp. Bot.* 50 265–276. 10.1016/S0098-8472(03)00037-6

[B24] Moxon S. Schwach F. Dalmay T. MacLean D. Studholme D. J. Moulton V. (2008). A toolkit for analysing large-scale plant small RNA datasets. *Bioinformatics* 24 2252–2253. 10.1093/bioinformatics/btn428 18713789

[B25] Ramachandran S. R. Mueth N. A. Zheng P. Hulbert S. H. (2020). Analysis of miRNAs in two wheat cultivars infected with *Puccinia striiformis f. sp. tritici*. *Front. Plant Sci.* 10:1574. 10.3389/fpls.2019.01574 31998329 PMC6965360

[B26] Ravichandran S. Ragupathy R. Edwards T. Domaratzki M. Cloutier S. (2019). MicroRNA-guided regulation of heat stress response in wheat. *BMC Genomics* 20:488. 10.1186/s12864-019-5799-6 31195958 PMC6567507

[B27] Safronova V. I. Stepanok V. V. Engqvist G. L. Alekseyev Y. V. Belimov A. A. (2006). Root-associated bacteria containing 1-aminocyclopropane-1-carboxylate deaminase improve growth and nutrient uptake by pea genotypes cultivated in cadmium supplemented soil. *Biol. Fertil. Soils* 42 267–272. 10.1007/s00374-005-0024-y

[B28] Su Y. Zhu Y. Lin A. Zhang X. (2005). Interaction between cadmium and atrazine during uptake by rice seedlings (*Oryza sativa* L.). *Chemosphere* 60 802–809. 10.1016/j.chemosphere.2005.04.022 15936797

[B29] Di Toppi L. S. Lambardi M. Pazzagli L. Cappugi G. Durante M. Gabbrielli R. (1998). Response to cadmium in carrot in vitro plants and cell suspension cultures. *Plant Sci.* 137 119–129. 10.1016/S0168-9452(98)00099-5

[B30] Turner D. A. (1985). Miranda: a non-strict functional language with polymorphic types. *Lect. Notes Computer.* 201 1–16. 10.1007/3-540-15975-4_26

[B31] Wang H. Wang H. Liu R. Xu Y. Lu Z. Zhou C. (2018). Genome-wide identification of TCP family transcription factors in *Medicago truncatula* reveals significant roles of miR319-targeted TCPs in nodule development. *Front. Plant Sci.* 9:774. 10.3389/fpls.2018.00774 29942322 PMC6004737

[B32] Wang S. Wong D. Forrest K. Allen A. Chao S. Huang B. (2014). Characterization of polyploid wheat genomic diversity using a high-density 90 000 single nucleotide polymorphism array. *Plant Biotechnol. J.* 12 787–796. 10.1111/pbi.12183 24646323 PMC4265271

[B33] Zhang X. Li K. Xing R. Liu S. Chen X. Yang H. (2018). miRNA and mRNA expression profiles reveal insight into chitosan-mediated regulation of plant growth. *J. Agric. Food Chem.* 66 3810–3822. 10.1021/acs.jafc.7b06081 29584426

[B34] Zhao X. Hong P. Wu J. Chen X. Ye X. Pan Y. (2016). The tae-miR408-mediated control of TaTOC1 genes transcription is required for the regulation of heading time in wheat. *Plant Physiol.* 170 1578–1594. 10.1104/pp.15.01216 26768600 PMC4775108

[B35] Zheng Y. Ding Y. Sun X. Xie S. Wang D. Liu X. (2016). Histone deacetylase HDA9 negatively regulates salt and drought stress responsiveness in *Arabidopsis*. *J. Exp. Bot.* 67 1703–1713. 10.1093/jxb/erv562 26733691

[B36] Zhou M. Zheng S. Li Y. Liu R. Zhang L. Wu Y. (2020). Comparative profiling of roots small RNA expression and corresponding gene ontology and pathway analyses for low- and high-cadmium–accumulating genotypes of wheat in response to cadmium stress. *Funct. Integr. Genomics* 20 177–190. 10.1007/s10142-019-00710-2 31435847

[B37] Zhou M. Zheng S. Liu R. Lu L. Zhang C. Zhang L. (2019). The genome-wide impact of cadmium on microRNA and mRNA expression in contrasting Cd responsive wheat genotypes. *BMC Genomics* 20:615. 10.1186/s12864-019-5939-z 31357934 PMC6664702

[B38] Zhou Q. Yang Y. Shen C. He C. Yuan J. Yang Z. (2017). Comparative analysis between low- and high-cadmium-accumulating cultivars of *Brassica parachinensis* to identify difference of cadmium-induced microRNA and their targets. *Plant Soil* 420 223–237. 10.1007/s11104-017-3380-0

[B39] Zhou Z. Song J. Yang Z. (2012). Genome-wide identification of *Brassica napus* microRNAs and their targets in response to cadmium. *J. Exp. Bot.* 63 4597–4613. 10.1093/jxb/ers136 22760473 PMC3421990

